# Motor phenotype and magnetic resonance measures of basal ganglia iron levels in Parkinson's disease^☆^

**DOI:** 10.1016/j.parkreldis.2013.08.011

**Published:** 2013-12

**Authors:** Nico Bunzeck, Victoria Singh-Curry, Cindy Eckart, Nikolaus Weiskopf, Richard J. Perry, Peter G. Bain, Emrah Düzel, Masud Husain

**Affiliations:** a Department of Systems Neuroscience, University Medical Center Hamburg-Eppendorf, Germany; b Institute of Cognitive Neuroscience, University College London, UK; c Institute of Neurology, University College London, UK; d Wellcome Trust Centre for Neuroimaging, Institute of Neurology, UCL, London, UK; e Imperial College London, UK; f Institute of Cognitive Neurology and Dementia Research, Otto-von-Guericke-University, Magdeburg, Germany; g German Centre for Neurodegenerative Diseases (DZNE), Standort Magdeburg, Germany; h Nuffield Department of Clinical Neurosciences, University of Oxford, UK; i Department of Experimental Psychology, University of Oxford, UK

**Keywords:** Parkinson's disease, Motor subtypes, Basal ganglia, Iron, Magnetic resonance imaging

## Abstract

**Background:**

In Parkinson's disease the degree of motor impairment can be classified with respect to tremor dominant and akinetic rigid features. While tremor dominance and akinetic rigidity might represent two ends of a continuum rather than discrete entities, it would be important to have non-invasive markers of any biological differences between them *in vivo,* to assess disease trajectories and response to treatment, as well as providing insights into the underlying mechanisms contributing to heterogeneity within the Parkinson's disease population.

**Methods:**

Here, we used magnetic resonance imaging to examine whether Parkinson's disease patients exhibit structural changes within the basal ganglia that might relate to motor phenotype. Specifically, we examined volumes of basal ganglia regions, as well as transverse relaxation rate (a putative marker of iron load) and magnetization transfer saturation (considered to index structural integrity) within these regions in 40 individuals.

**Results:**

We found decreased volume and reduced magnetization transfer within the substantia nigra in Parkinson's disease patients compared to healthy controls. Importantly, there was a positive correlation between tremulous motor phenotype and transverse relaxation rate (reflecting iron load) within the putamen, caudate and thalamus.

**Conclusions:**

Our findings suggest that akinetic rigid and tremor dominant symptoms of Parkinson's disease might be differentiated on the basis of the transverse relaxation rate within specific basal ganglia structures. Moreover, they suggest that iron load within the basal ganglia makes an important contribution to motor phenotype, a key prognostic indicator of disease progression in Parkinson's disease.

## 1 Introduction

Recent evidence suggests that motor-impairments in Parkinson's disease (PD) can be classified on a continuum with tremor dominant (TD) and akinetic rigid (AR) symptoms as extreme ends [1]. However, the precise underlying differences in neural pathology remain to be established. Physiologically, patients with predominantly AR phenotype have higher levels of neuronal loss, gliosis, extra-neuronal melanin deposits and neuro-axonal dystrophy in the SN compared to individuals with predominantly TD phenotype [2]. Furthermore, AR symptoms are associated with greater reductions in dopamine levels within the globus pallidus [3], and higher levels of cortical Lewy bodies [4]. Nigro-striatal degeneration in PD closely correlates with bradykinesia and rigidity but, importantly, *not* with tremor [5].

Another pathological hallmark of PD is alteration of brain iron level [6]. In particular, iron levels of the basal ganglia (BG), including substantia nigra (SN), are increased in PD pointing toward a dopamine-related dysfunction of the brain's iron homeostasis [6]. Moreover, work in animals [7] raises the possibility of a direct link between AR/TD motor symptoms and changes in iron levels within the BG.

In vivo, structural changes can be quantified on the basis of MRI measures such as R2* and magnetization transfer (MT) [8]. R2* is sensitive to iron levels [9] and in the midbrain it correlates with impaired motor performance [10]. MT, on the other hand, relates to the exchange of magnetization between mobile water protons and protons that are immobilized by macromolecules [11]. Although MT changes can have several region-specific physiological causes, within the SN of PD patients [12] and healthy older controls [13] MT changes likely relate to neuronal loss and/or degradation of the neuromelanin macromolecule scaffolding [14].

Here, we investigated the pathophysiology of PD and their relationship with motor symptoms using MRI. We predicted higher structural integrity (MT and/or size) of the BG in healthy controls compared to PD patients, and a direct relationship between iron content (R2*) and AR/TD motor symptoms. Finally, we investigated the relationship between MT and R2* within these structures [6].

## 2 Methods

### 2.1 MRI acquisition

Whole-head quantitative MRI was performed on a 3-tesla whole body magnetic resonance imaging scanner (Magnetom TIM Trio, Siemens). It comprised multi-parameter mapping, which was based on multi-echo 3D FLASH (fast low angle shot) acquisitions at a spatial resolution of 1 mm^3^ [8]. Briefly, whole brain images were acquired with predominant T1-, proton density, and MT-weighting imposed by the choice of repetition time (TR) and flip angle (T1-w: 18.7 ms/20°; PD-w and MT-w: 23.7 ms/6°) and by applying an off-resonance Gaussian-shaped RF pulse (4 ms duration, 220° nominal flip angle, 2 kHz frequency offset) prior to non-selective excitation for the MT-w acquisition, respectively. Alternating gradient echoes were acquired at six equidistant echo times (TE) between 2.2 ms and 14.7 ms for the T1- and MT-weighted acquisitions with two additional echoes at TE = 17.2 ms and 19.7 ms for the proton density-weighted acquisition. These multiple TE images were later averaged into one image for each weighting (e.g., T1-w, MT-w, PD-w) to increase signal to noise ratio.

Each scanning sequence (MTw, T1w, and proton density-weighted) was relatively short (only ∼7 min) which reduces possible within-scan motion. Between scan motion was accounted for by co-registration of the acquired images. To keep motion artifacts to a minimum, care was taken that each subject could rest on the scanner bed in a comfortable position and cushion padding was used for head fixation. Therefore, motion artifacts were minimized, which was further corroborated by visual inspection of the images.

### 2.2 Data processing and region of interest analysis

Data were reconstructed using SPM8 routines (www.fil.ion.ucl.ac.uk/spm) and Matlab tools (The Math-Works Inc.; Natick, MA, USA). For each subject, we calculated parameter maps of the MT saturation and the effective transverse relaxation rate R2* [8]; the images for different modalities were co-registered using rigid-body transformation. MT saturation can be regarded as a semi-quantitative measure, which corresponds to the percentage loss of magnetization imposed by a single MT pulse (in percent units – p.u). MT is implicitly corrected for differences in relaxation times and excitation flip angle, thus differing from the more commonly used MT ratio, the percentage reduction of the steady state signal. We did not use MT-ratio (MTR) since it is sensitive to non MT-specific effects such as changes in T1 or the RF transmit field inhomogeneities.

For each subject the following ‘Regions Of Interest’ (ROIs) were defined from native space (T1-image) using *FreeSurfer* (http://surfer.nmr.mgh.harvard.edu/): pallidum, putamen, caudate, nucleus accumbens and (entire) thalamus. Further subdivision of these structures was beyond the scope of this study. The thalamus was included since it constitutes a major input/output region of the BG and thus is of interest in the pathophysiology of PD. For each ROI we computed its volume, and mean R2* and MT value (Table S1).

**Fig. 1:**
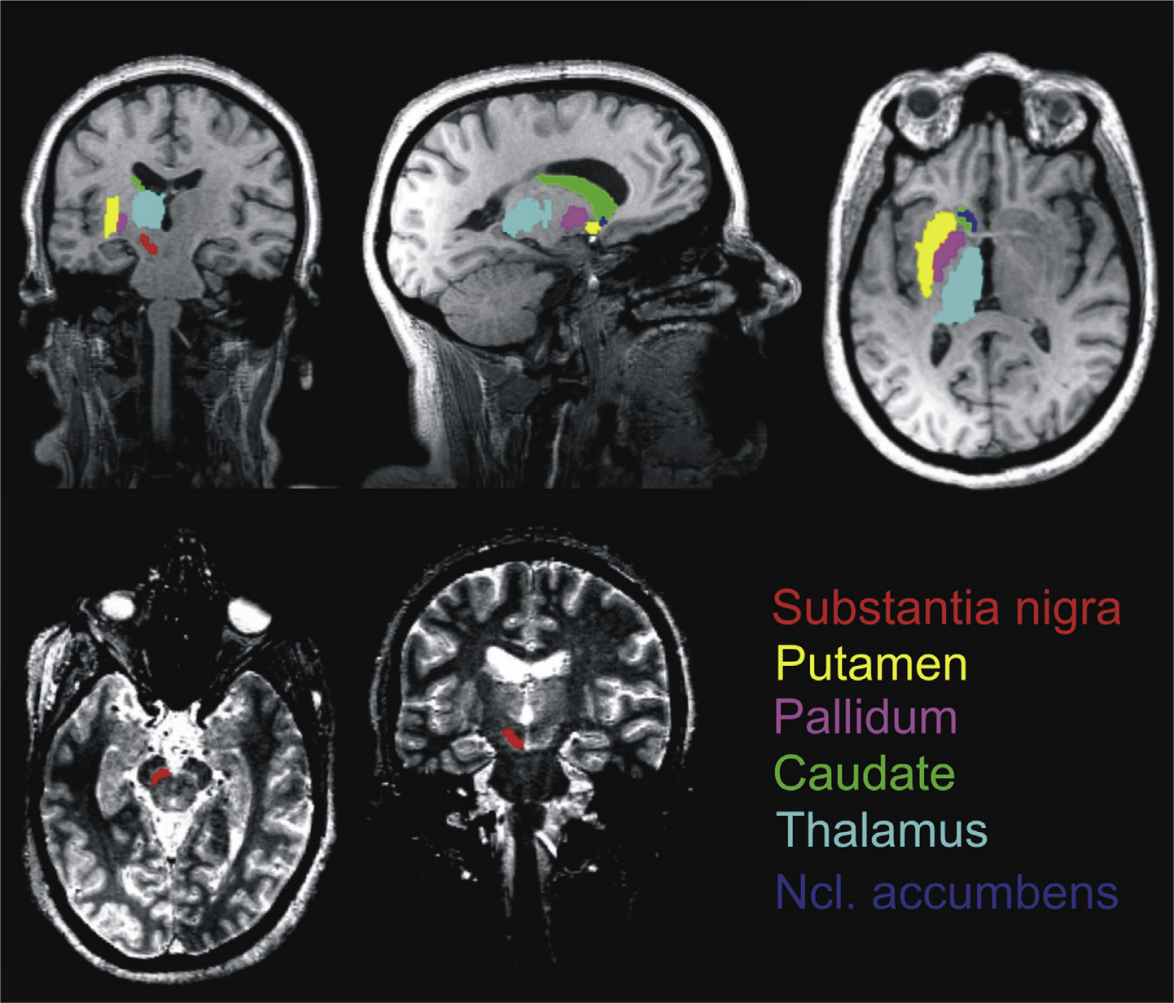
Regions of interest (ROI). The SN was defined manually for each subject. All other ROIs were segmented automatically (see Methods). Upper row shows left hemisphere ROIs overlaid on an individual T1-weighted image and lower row shows the subject's MT-image.

FreeSurfer does not support routines to automatically segment the SN. Therefore, it was defined manually for each subject on the basis of their MT-image (see Fig. 1) by a rater who was blind with respect to the subject's identity (C.E.). For a subset of 14 images this procedure was performed twice. An Intraclass Correlation Coefficient agreement (ICC agreement) of 0.90 for the right and 0.91 for the left hemisphere indicated high intra-rater reliability.

The SN was segmented on the basis of MT images for three reasons. First, on MT-images the SN can be distinguished from surrounding structures as a bright stripe while the adjacent red nucleus and cerebral peduncle appear dark (Fig. 1); second, we established this procedure in previous studies [13]; and third, it was demonstrated that MT parameter maps outperform T1-weighted images for SN segmentation [15]. In fact, the advantage of MT maps in contrast to T1-w images is that they are insensitive to iron susceptibility effects and thus mix fewer contrast components. Moreover, SN segmentation using T2*-w images might suffer from the problem that T2* contrast changes protrude beyond the structure with different susceptibility (i.e. SN).

FreeSurfer is one of the most reliable tools for automated segmentation currently available. Its morphometric procedures show good test-retest reliability across scanner manufacturers and field strengths [16]. Nevertheless, high accuracy for each ROI was confirmed by visual inspection. Rare cases of minor imprecision were not manually corrected.

### 2.3 Group comparisons

To identify possible global brain atrophies, individual whole brain measures were calculated for each modality (volume, MT and R2*) and compared using linear mixed effects (LME) models. Moreover, for each ROI volume, MT and R2* values were compared using LME models which included hemisphere as within factor. None of the contrasts of interest showed a significant interaction between diagnosis (PD vs. controls) and hemisphere. Models on volumetric measures included intracranial volume (ICV) as covariate of no interest to control for potential global brain atrophies. All statistical models included age as covariates of no interest to control for possible age-related effects.

### 2.4 Regression analyses

We used linear regression analysis to investigate:

(1) the relationship between brain measures (R2* and MT) and motor symptoms (i.e. subtype ratio; patients only),
(2) the correlation between MT and R2* values within each ROI (patients and controls).

Each linear regression analysis included two steps which both resulted in a $R_{\text{adj}}^{2}$ value (_adj_ abbreviates adjusted): first, a model was computed including only one variable of interest (e.g., MT) and the two covariates of no interest (age and ICV). Next, the second variable of interest (e.g., R2*) was added to the model. The difference between both $R_{\text{adj}}^{2}$ (i.e. $\Delta R_{\text{adj}}^{2}$ also known as ‘$R_{\text{adj}}^{2}$ added’) corresponds to the increase in explained variance by the second variable of interest [17]. Thus, it can be regarded as effect size for the correlation between both variables of interest independent of the variables of no interest (i.e. age, ICV). Note, that this type of linear regression analysis does not provide r-values.

All statistical analyses were conducted using ‘R’ (R Development Core Team, 2007; version 2.11.1). In the results section, the term ‘significant’ relates to *p*-values <0.05.

Note that we report individual *p*-values uncorrected for multiple comparisons. This approach is based on the suggestion that tests of a priori hypotheses (as in our case, see Introduction) do not necessarily need a significance level adjustment [18]. Instead, the number of observed significant results (four in the group comparisons and six in the regression analyses) exceed the number of probable random effects (0.9 for the regression analyses and group comparison each) which underlines the validity of our results [18].

## 3 Results

### 3.1 Participants: demographics and whole brain analysis

40 Subjects participated in the study: twenty PD patients (fulfilling Queens Square Brain bank criteria [19]) recruited from neurology clinics and twenty healthy control subjects. None of our patients had advanced PD with major signs of postural instability and gait difficulty [20], or any diagnosed psychiatric or neurological disorders other than PD. In particular, exclusion criteria involved impulse control disorder (ICD), depression (i.e. Beck Depression Inventory score ≥21) or cognitive impairments (i.e. Mini-Mental State Examination score < 25). Impulse control problems were assessed by the managing neurologist using appropriate questionnaires [21].

None of the healthy controls reported a history of neurological, psychiatric or medical disorders or any current medical problems. All subjects gave written informed consent in accordance with the approval of the local ethics committee (University College London, UK).

Motor subtype (i.e. subtype ratio, AR/TD) in PD patients was defined according to the ratio of UPDRS III tremor score and akinetic/rigid score [20]. AR/TD classification was made on the basis of current signs, with all patients on medication at time of assessment. Nevertheless, there was a high correlation between our scores [20] and scores as defined by Ref. [22] which is based on examination findings *and* history – and therefore includes symptoms reported OFF-state (*r* = 0.62, *p* = 0.004). Moreover, none of our patients were of mixed subtype, which might be more marked tremor responsive to dopaminergic medications [23].

Note that, for the analysis of our MR-data, the scale by Kang et al. [20] was chosen as primary scale over others (i.e. Ref. [22]) because none of our patients showed any major symptoms of postural instability and gait difficulty.

Patients and controls were matched regarding age, gender ratio, whole brain volume, intracranial volume (ICV) and mean MT and R2* (all *p*-values >0.21). In PD-patients, subtype ratio did not correlate with whole brain volume (*t*(17) = 0.609, *p* = 0.551), ICV (*t*(17) = 0.772, *p* = 0.451), or any other measure such as time of symptom onset or l-dopa equivalent unit (all *p*'s > 0.18). l-dopa equivalence units were quantified as in Ref. [24].

According to Ref. [20] our PD sample comprised 10 AR and 10 TD patients which differed only in subtype ratio (*p* < 0.0001), but no other variables of interest (Table 1). Moreover, AR and TD did not differ from healthy controls regarding any measure of interest (e.g., age, gender ratio, etc.; see Table 1, all *p* > 0.05) and there were no significant outliers in any group.

### 3.2 Region of interest (ROI) analysis

#### 3.2.1 Substantia nigra (SN)

**Fig. 2:**
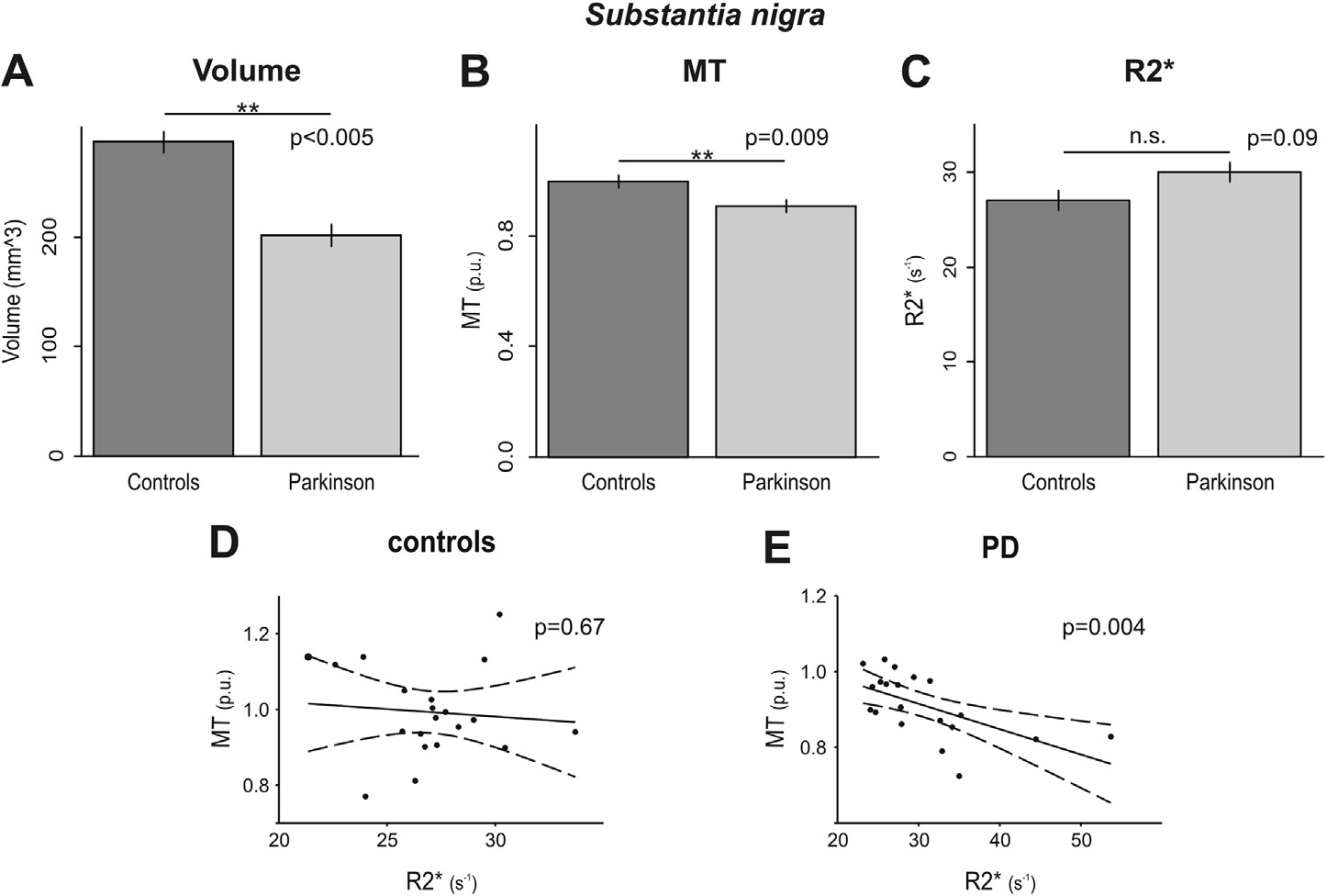
Controls had larger SN volumes (A) and higher MT values (B). R2* and MT correlated negatively in PD-patients (D) but not controls (C). Bar-plots are based on LME parameter estimates and scatter-plots are based on individual raw data (see Methods).

PD patients exhibited significantly smaller SN volumes (*F*(1,36) = 9.51, *p* = 0.004) (Fig. 2A) and lower MT values (*F*(1,37) = 7.67, *p* = 0.009) compared to healthy controls (Fig. 2B). There were no significant differences in R2* (*p* = 0.09, Fig. 2C). Further analyses of R2* of the medial and lateral SN also did not reveal any significant group differences (*p* > 0.05) or interactions between group and laterality (i.e. group specific differences in the medial or lateral part of the SN; *p* > 0.31). See Table S1 for a summary.

When relating both MT and R2* of the SN by using regression analysis (see Methods), we observed a significant negative correlation across the whole sample, including controls and PD patients (*F*(1,35) = 6.90, *p* = 0.01). Thus, the lower MT – and, by inference, reduced structural integrity of the SN – the higher R2* (i.e. iron).

Importantly, the analysis also revealed a significant interaction with group (*F*(1,35) = 11,01, *p* = 0.002) indicating statistically significant differences between healthy controls and PD patients. Indeed, a significant correlation between R2* and MT was found only in the 20 PD patients (*t*(17) = −3,34, *p* = 0.004; Fig. 2E; Table S2), but not in healthy controls (*p* = 0.67; Fig. 2D). There were no other statistically significant effects (e.g., with *subtype ratio*).

#### 3.2.2 Putamen

**Fig. 3:**
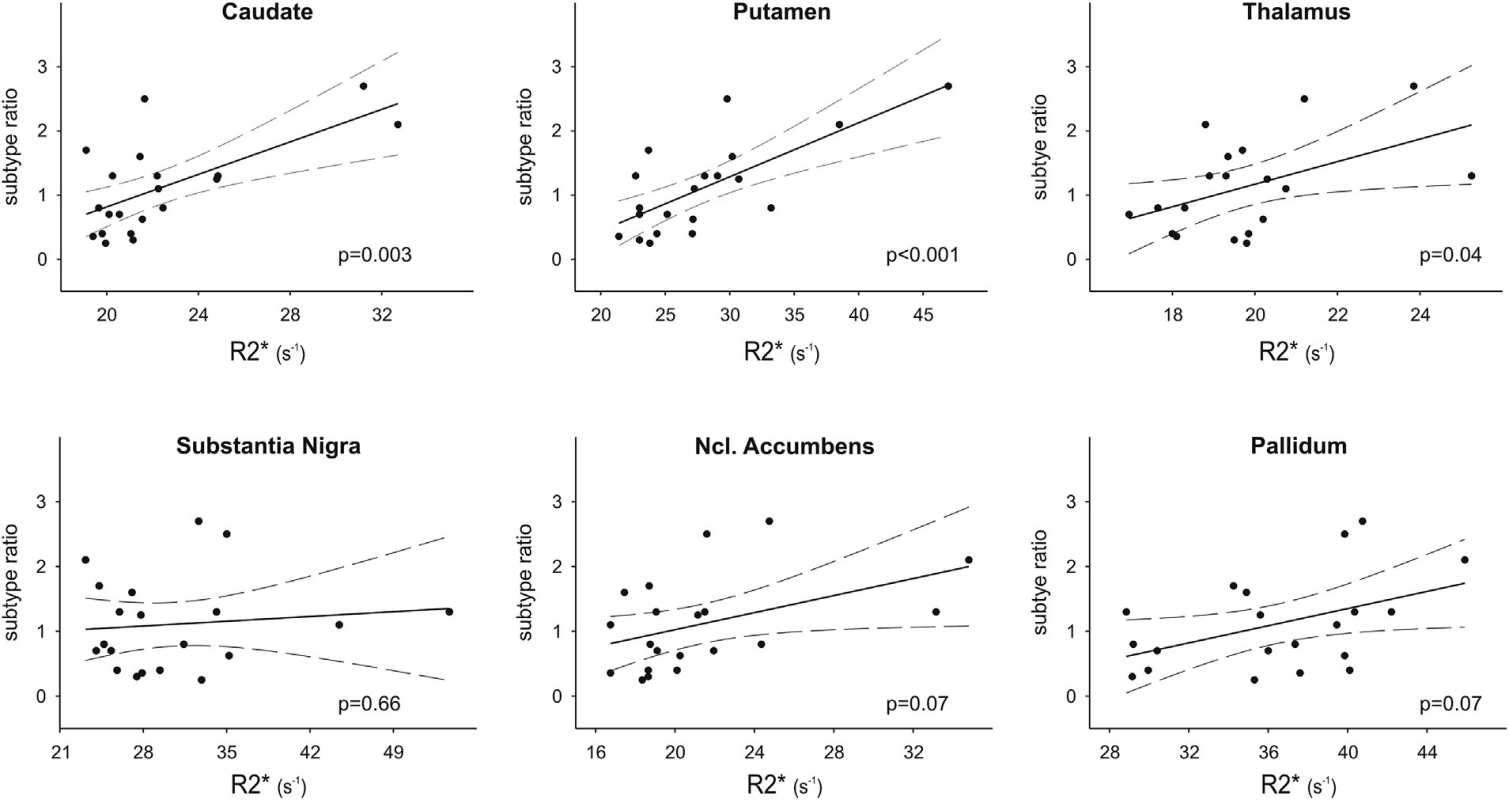
R2* and subtype ratio correlations. Within the putamen, caudate and thalamus, but not substantia nigra, ncl. accumbens and pallidum, subtype ratio was positively correlated with R2*; the greater the tendency towards the TD motor phenotype the higher R2*. Note that the two subjects corresponding to extreme data points in the caudate, putamen and thalamus did not differ in any dimension other than R2* within these *specific* BG structures (i.e. they were no outliers regarding whole brain volume, intracranial volume, whole brain R2*, whole brain MT, UPDRS, ACE-R, Barratt Impulsiveness Scale, time of symptom onset, age and medication). Thus, they were not excluded from the analyses.

Putamen volumes were larger in healthy controls vs. PD patients (*F*(1,36) = 5.55, *p* = 0.02). There were no significant differences in MT (*p* = 0.35) or R2* (*p* = 0.7) – see Table S1. However, regression analysis across all 20 PD patients revealed a significant positive correlation between R2* and subtype ratio (t(17) = 4.342, *p* = 0.0004) (Fig. 3). Thus, the higher the ratio (>1 defines the TD group; see above) the greater R2*.

Regression analysis using MT and R2* of the putamen revealed a significant correlation between both measures across the whole sample (*t*(37) = −2.78, *p* = 0.008), which –again – was driven by a significant negative correlation between MT and R2* in the 20 PD patients (*t*(17) = −2.21, *p* = 0.04) but not in controls (*p* = 0.13) (Table S2).

#### 3.2.3 Caudate

There was no difference in volume (*p* = 0.33), R2* (*p* = 0.85) or MT (*p* = 0.58) between patients and controls (Table S1). However, across the 20 PD patients there was a significant positive correlation between R2* and subtype ratio (*t*(17) = 3.416, *p* = 0.003) (Fig. 3). There were no other significant effects (Table S2).

#### 3.2.4 Pallidum

There was no significant difference in volume (*p* = 0.07) or R2* (*p* = 0.39) but PD patients exhibited lower MT values than healthy controls (*F*(1,37) = 5.004, *p* = 0.03; Table S1). There were no other statistically significant effects (Table S2).

#### 3.2.5 Thalamus

As for putamen and caudate, there was no significant group effect on volume (*p* = 0.51), R2* (*p* = 0.6) or MT (*p* = 0.17; Table S1) but a significant linear relationship (across the 20 PD patients) between subtype ratio and R2* (*t*(16) = 2.197, *p* = 0.04) (Fig. 3). There were no other significant effects (*p* > 0.05) (Table S2).

#### 3.2.6 Nucleus accumbens

There was no significant group difference in volume (*p* = 0.56), R2* (*p* = 0.23) or MT (*p* = 0.46; Table S1) but a positive relationship between subtype ratio and MT (*t*(17) = 2.27, *p* = 0.04) across the 20 PD patients. No other significant effects were observed (*p* > 0.05).

### 3.3 Summary

Taken together, three main patterns emerged:

(1) In PD patients the SN was characterized by reduced volume and lower MT values (Fig. 2, Table S1),
(2) Within the putamen, caudate and thalamus R2* correlated positively with subtype ratio (Fig. 3), so the greater the tendency towards the TD motor phenotype the higher R2* (i.e. iron),
(3) Within the SN and putamen R2* correlated negatively with MT in PD patients but *not* in healthy controls (Table S2).

## 4 Discussion

Our results point towards a functional link between brain iron accumulation and PD motor symptoms. They show that MRI parameters can demonstrate differences in the expression of AR and TD symptoms, and that R2* in some BG structures correlates with motor phenotype. Therefore, this technique could provide a non-invasive biomarker to assess trajectories of disease and responses to treatment in trials, as well as potentially providing mechanistic insights into the heterogeneity within the PD population.

### 4.1 R2* and motor subgroup

Our findings of reduced SN volumes and MT values in PD patients (Fig. 2) are consistent with previous MRI studies [12,25]. They correspond well to the known pathophysiology of PD, i.e. neuronal loss and gliosis [19], and the notion that decreases in SN MT might reflect these structural changes. Although we did not detect any significant correlations with motor symptoms and MT, R2* or volume, respectively, within the SN, there was a close relationship between R2* and motor subtype in the putamen, caudate and thalamus (Fig. 3).

Increased R2* within the BG (including SN) and thalamus of PD patients has been described previously and interpreted as evidence of enhanced iron levels [9]. However, these reports contrast with findings of absent changes in nigral iron levels in PD [26,27]. One possibility is that enhanced BG iron levels are not a general feature of PD but might in fact relate to *motor phenotype*. Our results directly support this assumption by showing no overall differences in R2* between PD patients and healthy controls, but importantly a significant correlation between R2* and subtype ratio within the putamen, caudate and thalamus. It suggests that the amount of iron accumulation predicts severity of *tremor dominant* symptoms. Conversely, the lower R2*, the more pronounced the akinetic rigid symptoms. Therefore, R2* appears to be a potential biomarker for motor phenotype in PD which might help to improve strategies in medical care even in the early stages of the disease [28].

It might seem surprising that there was no correlation within the SN between motor phenotype and either volume, R2* or MT. However, it is possible that SN degeneration is common to all sub-groups, but factors in other parts of the basal ganglia distinguish between phenotypes. For example, it has been demonstrated that AR patients exhibit greater reductions of dopamine in the globus pallidus [3]. Future post mortem studies might help in establishing which pathological features are common and different between motor phenotypes.

### 4.2 Basal ganglia iron in Parkinson's disease

Although iron levels seem to be associated with PD [29] the precise pathophysiological mechanisms are still a matter of debate [6,27]. One possibility is that imbalanced iron concentration trigger oxidative stress, which leads to nigral cell loss and subsequently results in PD motor symptoms. However, this causative relationship has been challenged by the view that enhanced iron levels might only be a secondary phenomenon [30].

While our data cannot clarify the causal role of iron in PD, they clearly show that the relationship between iron levels (R2*) and motor symptoms (i.e. subtype ratio) is not a general effect across all BG structures. Rather, it seems to be predominantly expressed within the caudate, putamen, and inter-connected thalamus (Fig. 3). In addition, the effect seems to be iron specific rather than a non-specific degenerative phenomenon because there was no correlation between subtype ratio and MT (except nucleus accumbens) in any ROI.

It might perhaps be surprising that increased R2* (i.e. iron load) is most marked in patients with TD rather than AR symptoms, given the generally worse prognosis in the latter. Patients with predominant AR phenotype have higher levels of cortical Lewy bodies [4], consistent with the finding that this subgroup is more likely to develop cognitive dysfunction [31]. They also have higher levels of neuronal loss, gliosis, extra-neuronal melanin deposits and neuro-axonal dystrophy in the SN [2] and exhibit greater reductions in dopamine level in the internal globus pallidus [3]. Our findings consistently show that enhanced R2* scales with TD symptoms across several BG structures which is in line with previous animal studies. In rabbits and monkeys, systemic (intravenous) injections of iron in the form of dextran lead to increased iron deposits in the BG, with tremor – not rigidity – as the motor phenotype [7]. Thus, iron load is likely to be one factor out of many that affect the phenotype of PD patients [1].

Another differentiating feature between PD patients and healthy controls lies in the relationship between R2* and MT (Table S2). In controls, both measures did not correlate in any ROI which seems to be characteristic for the healthy brain, perhaps reflecting a balanced iron homeostasis [6]. In PD patients, on the other hand, iron content (R2*) varied negatively as a function of structural integrity (MT) within the SN and putamen (Table S2). This pattern conforms to the notion that enhanced iron levels relate to neurodegeneration and motor symptoms of PD [6].

Finally, it should be noted that our results are not based on a direct comparison between AR vs. TD subgroups but regression analyses including all 20 PD patients and their AR/TD subtype ratio. This approach rests on the notion that TD and AR subtypes reflect different points on a continuum. Indeed, some patients who initially present with one motor phenotype can later develop features of the other (albeit predominantly from TD to AR, rather than the reverse) [32] which argues against AR and TD being two discrete entities.

### 4.3 Conclusion

Overall, our findings suggest that AR and TD motor symptoms of PD directly relate to R2* within specific BG structures and thalamus. This result is important because emerging evidence suggests that patients with predominant AR phenotype are more likely to develop cognitive impairment, falls, sleep disorders and autonomic impairment. If R2* is indeed a good marker of iron load, this points to an important pathophysiological marker to distinguish between motor phenotypes, and hence disease prognosis.

## Competing interests

None.

## Funding

This work was supported by the Wellcome Trust and NIHR BRC at UCL/UCLH and the Hamburg state cluster of excellence (neurodapt!).

## Figures and Tables

**Table 1:** Group comparison. AR and TD did not differ in age (*p* = 0.98), gender ratio (*p* = 0.99), overall scores in UPDRS (*p* = 0.29), Addenbrooke's Cognitive Examination-Revised (ACE-R; *p* = 0.42), Barratt Impulsiveness Scale (BIS; *p* = 0.87), time of symptom onset (*p* = 0.63) and medication (*p* = 0.19). AR and TD did not differ from healthy controls (all *p* > 0.05) regarding age, intracranial volume (ICV), whole brain volume and gender.

	Controls	PD patients	AR	TD
*n*	20	20	10	10
Mean age (SD; range)	66.0 (9.1; 43–85)	66.25 (9.0; 42–84)	66.3 (5.9; 58–79)	66.2 (11.7; 42–84)
Gender ratio: male/female	10/10	11/9	5/5	6/4
Time symptom onset in years (SD)		6.27 (4.4)	6.75 (5.0)	5.78 (3.8)
Unified Parkinson Disease Rating Scale (UPDRS)		34.6 (17.4)	30.4 (13.2)	38.8 (20.6)
Subtype ratio		1.1 (0.7)	0.5 (0.2)**	1.7 (0.6)**
Addenbrooke's Cognitive Examination-Revised (ACE-R)		88.4 (15.0)	91.3 (7.5)	85.6 (20.1)
Barratt Impulsiveness Scale (BIS)		0.5 (0.1)	0.5 (0.1)	0.5 (0.1)
l-dopa equivalence units		393.8 (339.0)	375.4 (268.2)	412.2 (412.2)
Whole brain volume in mm³ (SD)	1235.3 (146.2)	1230.4 (102.1)	1193.3 (101.1)	1267.4 (93.5)
Intracranial volume mm^3^ (SD)	1647.7 (201.5)	1705.1 (140.6)	1678.1 (153.8)	1732.0 (128.4)
Whole brain MT (SD)	0.833 (0.14)	0.848 (0.09)	0.835 (0.08)	0.861 (0.09)
Whole brain R2* (SD)	22.4 (0.9)	22.9 (2.0)	23.1 (2.5)	22.8 (1.6)

**Denotes statistically significant difference (*p* < 0.001).
