# Supplementary material for: Motor phenotype and magnetic resonance measures of basal ganglia iron levels in Parkinson's disease
Source: Parkinsonism Relat Disord. 2013 Dec;19(12):1136–42. doi: 10.1016/j.parkreldis.2013.08.011 (PMC3878384; doi:10.1016/j.parkreldis.2013.08.011)
Supplement: Supplementary file 1 [file mmc1.docx]

**Table S1**. Volume, MT and R2* data for all regions of interest and groups. Abbreviations: M – mean, SD – standard deviation, p.u. – percent units.

|  |  | Controls |  | PD patients | | AR |  | TD |  |
| --- | --- | --- | --- | --- | --- | --- | --- | --- | --- |
|  |  | M | SD | M | SD | M | SD | M | SD |
|  |  |  |  |  |  |  |  |  |  |
| **Substantia Nigra:** | | |  |  |  |  |  |  |  |
| Volume (mm^3^) | left | 286.0 | 61.5 | 201.5 | 63.7 | 198.2 | 39.2 | 204.7 | 83.7 |
|  | right | 281.4 | 71.7 | 207.4 | 53.9 | 195.9 | 47.0 | 218.8 | 60.3 |
| MT (p.u.) | left | 1.00 | .13 | .91 | .09 | .92 | .09 | .89 | .11 |
|  | right | .99 | .11 | .91 | .08 | .93 | .07 | .90 | .09 |
| R2* (s^-1^) | left | 26 | 3 | 30 | 8 | 27 | 4 | 32 | 10 |
|  | right | 28 | 3 | 31 | 8 | 29 | 4 | 34 | 10 |
|  |  |  |  |  |  |  |  |  |  |
| **Putamen:** |  |  |  |  |  |  |  |  |  |
| Volume (mm^3^) | left | 4749.1 | 664.7 | 4678.5 | 717.7 | 4555.5 | 645.2 | 4801.5 | 798.5 |
|  | right | 4634.6 | 575.0 | 4431.3 | 618.4 | 4391.3 | 568.1 | 4471.3 | 693.6 |
| MT (p.u.) | left | .93 | .06 | .92 | .08 | .91 | .08 | .93 | .09 |
|  | right | .93 | .07 | .90 | .07 | .91 | .07 | .89 | .08 |
| R2* (s^-1^) | left | 28 | 5 | 27 | 5 | 25 | 3 | 30 | 6 |
|  | right | 29 | 5 | 29 | 7 | 25 | 4 | 32 | 8 |
|  |  |  |  |  |  |  |  |  |  |
| **Ncl. Caudatus:** | |  |  |  |  |  |  |  |  |
| Volume (mm^3^) | left | 3526.1 | 511.1 | 3505.3 | 388.8 | 3408.3 | 417.1 | 3602.2 | 352.7 |
|  | right | 3338.6 | 476.4 | 3332.2 | 504.9 | 3229.2 | 525.4 | 3435.1 | 488.4 |
| MT (p.u.) | left | .83 | .08 | .86 | .08 | .85 | .08 | .87 | .09 |
|  | right | .83 | .08 | .82 | .06 | .79 | .05 | .85 | .06 |
| R2* (s^-1^) | left | 22 | 2 | 23 | 3 | 21 | 1 | 25 | 4 |
|  | right | 22 | 2 | 22 | 4 | 20 | 2 | 24 | 5 |
|  |  |  |  |  |  |  |  |  |  |
| **Pallidum:** |  |  |  |  |  |  |  |  |  |
| Volume (mm^3^) | left | 1484.1 | 236.0 | 1419.1 | 159.3 | 1368.4 | 109.4 | 1469.8 | 189.5 |
|  | right | 1345.1 | 203.6 | 1311.5 | 203.2 | 1242.9 | 205.1 | 1380.0 | 186.1 |
| MT (p.u.) | left | 1.22 | .10 | 1.15 | .12 | 1.14 | .13 | 1.16 | .13 |
|  | right | 1.20 | .10 | 1.15 | .13 | 1.17 | .14 | 1.13 | .12 |
| R2* (s^-1^) | left | 37 | 6 | 36 | 5 | 35 | 5 | 37 | 5 |
|  | right | 38 | 6 | 37 | 6 | 34 | 5 | 39 | 5 |
|  |  |  |  |  |  |  |  |  |  |
| **Thalamus:** |  |  |  |  |  |  |  |  |  |
| Volume (mm^3^) | left | 6409.3 | 745.3 | 6511.2 | 669.8 | 6431.0 | 677.4 | 6591.4 | 688.4 |
|  | right | 6862.9 | 914.7 | 6912.9 | 791.4 | 6806.6 | 750.7 | 7019.1 | 856.4 |
| MT (p.u.) | left | 1.03 | .08 | 1.02 | .07 | 1.03 | .06 | 1.02 | .08 |
|  | right | 1.06 | .08 | 1.02 | .07 | 1.01 | .06 | 1.02 | .08 |
| R2* (s^-1^) | left | 19 | 1 | 20 | 2 | 19 | 1 | 21 | 2 |
|  | right | 20 | 1 | 20 | 2 | 19 | 1 | 21 | 2 |
|  |  |  |  |  |  |  |  |  |  |
| **Ncl. Accumbens:** | |  |  |  |  |  |  |  |  |
| Volume (mm^3^) | left | 510.8 | 133.1 | 472.4 | 122.8 | 465.3 | 126.1 | 479.5 | 125.9 |
|  | right | 516.4 | 68.09 | 551.1 | 87.38 | 512.4 | 96.72 | 589.8 | 58.60 |
| MT (p.u.) | left | .75 | .05 | .75 | .05 | .73 | .04 | .77 | .05 |
|  | right | .79 | .05 | .81 | .06 | .79 | .05 | .83 | .07 |
| R2* (s^-1^) | left | 20 | 6 | 23 | 7 | 21 | 5 | 24 | 8 |
|  | right | 19 | 3 | 20 | 4 | 19 | 3 | 22 | 5 |
