# Supplementary material for: Motor phenotype and magnetic resonance measures of basal ganglia iron levels in Parkinson's disease
Source: Parkinsonism Relat Disord. 2013 Dec;19(12):1136–42. doi: 10.1016/j.parkreldis.2013.08.011 (PMC3878384; doi:10.1016/j.parkreldis.2013.08.011)
Supplement: Supplementary file 2 [file mmc2.docx]

**Table S2.** MT and R2* correlations in all ROIs. ΔR²_adj_ represents the effect size for the correlation (see methods), n.s.: not significant (p > 0.05). The direction of the correlations is indicated by the sign of the t-statistics.

|  | **Controls** | **PD patients** |
| --- | --- | --- |
| **SN** | n.s.  t(17)=-0.44, p=.67 | *t(17)=-3.34, p=0.004;*  *ΔR²_adj_=0.38* |
| **Putamen** | n.s.  t(17)=-1.58, p=0.13 | *t(17)=-2.21, p=0.04;*  *ΔR²_adj_=0.15* |
| **Pallidum** | n.s.  t(17)=-0.09, p=0.93 | n.s.  t(17)=-0.39, p=0.70 |
| **Thalamus** | n.s.  t(17)=-0.01, p=0.99 | n.s.  t(17)=0.96, p=0.35 |
| **Ncl. Accumbens** | n.s.  t(17)=1.60, p=0.13 | n.s.  t(17)=1.05, p=0.31 |
| **Caudate** | n.s.  t(17)=0.05, p=0.96 | n.s.  t(17)=-0.08, p=0.94 |
